# Disease-free survival after robotic-assisted laparoscopic total pelvic exenteration for recurrent cervical adenocarcinoma: A case report

**DOI:** 10.1097/MD.0000000000011611

**Published:** 2018-07-27

**Authors:** Qiyu Yang, Junying Tang, Lin Xiao

**Affiliations:** Department of Gynecology, Chongqing Medical University First Affiliated Hospital, Chongqing, China.

**Keywords:** cervical adenocarcinoma, robotic-assisted surgery, total pelvic exenteration

## Abstract

**Rationale:**

Pelvic exenteration is considered a method to treat central recurrent or persistent gynecologic malignancy after the initial therapy. The postoperative survival rate has been greatly increased by the improvement in the surgical technology and the perioperative management. Yet various complications are still impacting the quality of life. New technologies such as robotic surgery system made it possible to approach radical surgical resection by using a minimally invasive method.

**Patient concerns:**

The patient is a 53-year-old female with the cervical adenocarcinoma pelvic recurrence who had undergone the adjuvant chemo-radiotherapy and the laparoscopic radical hysterectomy in our hospital 2 years ago. She still expected her life to be prolonged through surgery therapy.

**Diagnoses:**

Locoregional recurrence of cervical adenocarcinoma.

**Interventions:**

A robotic total pelvic exenteration with ileal neobladder was performed.

**Outcomes:**

The postoperative results were excellent and after 17-month follow-up, the patient is alive and satisfied without any recurrence or distant metastasis.

**Lessons:**

For the patients with advanced or recurrent cervical cancer who are willing to receive surgical therapy and not sensitive to chemo-radiotherapy, robotic-assisted laparoscopic total pelvic exenteration is technically a feasible surgical method for recurrent pelvic malignancies. Yet the operation time should be further controlled to reduce complications which include pressure sore and thrombus. Moreover, appropriate assessment is required in the selection of the methods for reconstruction.

## 1 Introduction

Pelvic exenteration (PE), with an aim to complete en bloc resection of malignant lesions and pelvic organs, provides a potential treatment for the patients with advanced and recurrent pelvic malignancies.^[1]^ As the surgical technology and the perioperative management have been advanced, the postoperative survival rate has increased from 20% to approximately 60%.^[2]^ Complete surgical resection of all the lesions is critical for the patients to expect a longer survival span, whereas it challenges the surgeons to reduce the surgical complications, especially for patients who received the preoperative chemoradiotherapy, which increases tissue fragility.^[3]^

The Da Vinci Robot–assisted laparoscopic surgery refers to the developed traditional laparoscopic technique that bases on 3D vision, human arm simulation system, and tremor filtration system. Thus, it could complete the complex and precise operations by using a manipulation that is more flexible than human hands. This technique can accurately return to the last operation area and prevent accidental injury through the software preset and memory function,^[4]^ which makes it more preferable for a complex and long-time surgery like PE.

## 2 Case report

A 53-year-old female is reported, having been admitted in our Department of Gynecology for postcoital vaginal bleeding and diagnosed with cervical adenocarcinoma based on the pathological biopsy, with an International Federation of Gynecology and Obstetrics stage of IIB. She also has had the diabetes and hypertension for more than 2 years. In addition, her blood glucose and pressure were within the normal limits under the treatment of insulin, metformin, and irbesartan. The patient had received the laparoscopic radical hysterectomy after 4 courses of chemotherapy and 25 courses of radiotherapy (GTV DT54Gy/25f/5w, CTV DT46Gy/25f/5w, PTV DT46Gy/25f/5w). After the surgery, 2 courses of chemotherapy had been given. Two years later (in 2016), the patient developed an abnormal vaginal bleeding, with the biopsy of vaginal cuff finding adenocarcinoma tissue. The magnetic resonance imaging and Positron Emission Tomography-Computed Tomography (PET-CT) showed a locoregional recurrence of 3 × 4 cm in vaginal cuff, which invaded the urinary bladder and the rectum. Unfortunately, the patient was so upset, making her last will in her room.

After a discussion of multidisciplinary treatment, a robotic-assisted laparoscopic total pelvic exenteration (RALTPE) was performed with the DaVinci SI system (Intuitive Surgical Inc, Sunnyvale, CA) on November 15, 2016, by a combined approach with gynecology, urology, and gastrointestinal surgery.

First and foremost, a carbon dioxide pneumoperitoneum of 12 mm Hg was established and the robotic system was docked. Five ports (3 for 12 mm and 2 for 8 mm) were applied for the camera and other instruments. After the first separation of the adhesive tissues in pelvic cavity and the isolation of the vessels and the ureters on both sides, the paravesical and pararectal spaces were developed in a retroperitoneal method. Next, the vessels were clipped from the roots, and rectal resection and radical cystectomy were performed. Then, the incision of all the organs and fascias along basin sidewall was performed. Thus, the en bloc dissection was completed.

Afterward, an anastomosis (by a stapler of Johnson No. 29) was made between residual rectum and sigmoid colon, which was 3 cm above the anal verge. A 20 cm part of ileum with vascular pedicle was mobilized at 25 cm away from the ileocecal region, and a side-to-side ileoileostomy was performed to recover the digestive continuity. The proximal part of the isolated ileum was sutured to form a neobladder. The 2 ureters were then implanted in the ileal neobladder with 4-0 Vicryl, and they were supported by 2 ureteral stents.

Finally, the distal part of neobladder was exteriorized as an output duct by 2-0 Vicryl using the ileostomy at right lower abdomen, with an F16 drainage tube indwelt in the neobladder. One abdominal drainage tube and one pelvic drainage tube were placed. In addition, an anal canal was indwelt.

The whole operation continued for 700 minutes, with an estimated blood loss (EBL) of 300 mL. Before returning to the department of gynecology, the patient stayed in Intensive Care Unit for 12 hours. The pathological results confirmed the recurrence with malignant cells that invaded the bladder and the rectum. In addition, the margins were negative. On the eighth day, a rectosigmoid anastomotic fistula was discovered, and a double-barrel Transversostomy was performed promptly. The patient was discharged on the 37th day. Three months later the 2 ureteral stents were removed without dysuria or other complications. After 17-month follow-up, the patient is alive and satisfied without any recurrence or distant metastasis.

## 3 Discussion

Since reported by Brunschwig in 1948,^[5]^ the PE, inclusive of total pelvic exenteration, anterior pelvic exenteration, and posterior pelvic exenteration, has become an important method to treat the pelvic malignancies, especially for the advanced and recurrent cervical cancer. The preoperative chemoradiotherapy is an indispensable method to increase the opportunity of surgery. Yet it also leads to many irreversible complications, which to some extent increases the difficulty of surgery. For the patient with cervical adenocarcinoma in our case, who was less susceptible to radiotherapy and chemotherapy, the surgery was even more important and difficult. In addition, the patient had diabetes and hypertension, which was adverse to postoperative recovery. As a result, the method of surgery and the intraoperative coordination were of huge importance.

Initially, PE was performed using the open approach. Although the laparoscopic PE is feasible, it is not characterized by the preferable exposure and visualization of the operating field, superior ergonomics for the surgeon, and enhanced dexterity that robotic system provides.^[6]^ The robotic surgery offers safer and more stable operation as compared with the laparotomy and traditional laparoscopy, and thus reducing the incidence of complications.^[4]^ In our case, thanks to the accurate surgical manipulation in surgery, with EBL of 300 mL, the patient had no blood transfusion during the hospitalization. Yet the robotic systems come with certain disadvantages. Because the robotic devices are heavy and bulky, there is a lack of tactile perception and tensile feedback, and time consumption during the docking and undocking of the robot. In addition, the crowding of the operating theaters can occur with the relatively cumbersome robotic arms,^[7]^ especially for surgical assistants, who have to assist operators with their arms confined between the robotic arms. Fortunately, all these disadvantages can be overcome by the clear advantages of the enhanced visualization. In addition, as we overcame the learning curve with experience and prevented collisions by properly positioning the robotic ports, the operation time decreased.

For all the 21 patients with cervical malignancy received the robotic PE in the relevant literature, only 4 were for RALTPE.^[8–11]^ It is a consensus that the surgery is of small significance for patients with distant metastases,^[2]^ yet different medical centers are inconsistent in the indications of PE for the recurrent cervical cancer. Further researches are required to build a standard treating system.

For urinary reconstruction, 3 different methods are available, which include Bricker (incontinent cutaneous diversion), Miami (continent cutaneous diversion), and orthotopic neobladder. All the noted complications, that is, perineal abscess Miami's fistula, pyelonephritis, ureteral stenosis, and renal insufficiency, were reported in the cases that have received the Miami reconstruction.^[12,13]^ It was reported that the Miami was more controllable and more complex, with a higher incidence rate of late complications as compared with Bricker (19%–33% for dysuresia), whereas the 2 methods were reported with a similar rate of early complications. The orthotopic neobladder construction provides better quality of life, with a higher rate (12.5%) of postoperative complications.^[14,15]^

For the digestive tract reconstruction after resection of rectum, the standard method is the left colostomy. Direct anastomosis can increase the quality of life, yet with a higher incidence of severe complications, among which the anastomotic fistula occurs most frequently.^[16]^ Lawande et al have performed a successful coloanal anastomosis for a 35-year-old patient, without any early or late complications.^[14]^ In our case, a rectosigmoid anastomotic fistula occurred 8 days after the anastomosis, which was then treated by the reoperation. The history of diabetes, repeated preoperative chemoradiotherapy, history of pelvic surgery, and the low anastomosis site were deemed as the possible reasons for the leakage. In conclusion, patients should be selected prudently to perform a direct anastomosis, especially the patients with a history of preoperative chemoradiotherapy.

In a word, our case is another attempt to perform a RALTPE for a patient of recurrent cervical adenocarcinoma, with the treatment history of surgery, chemotherapy, and radiotherapy. A satisfactory postoperative result was obtained. It was concluded that RALTPE is technically a feasible surgical method for the recurrent pelvic malignancies, probably with lower blood loss and fewer complications than traditional surgery. Yet many other factors could affect the operative outcome, which include the technique level of the surgeons and the perioperative management in different hospitals. Thus, further comparative researches with large sample are required to draw a more comprehensive and reliable conclusion.

## Author contributions

**Conceptualization:** Qiyu Yang, Junying Tang.

**Data curation:** Qiyu Yang.

**Formal analysis:** Qiyu Yang, Lin Xiao.

**Investigation:** Qiyu Yang, Junying Tang, Lin Xiao.

**Methodology:** Junying Tang.

**Resources:** Junying Tang, Lin Xiao.

**Software:** Qiyu Yang, Lin Xiao.

**Supervision:** Junying Tang.

**Validation:** Lin Xiao.

**Visualization:** Qiyu Yang.

**Writing – original draft:** Qiyu Yang.

**Writing – review and editing:** Junying Tang, Lin Xiao.
